# Acupotomy Therapy for Chronic Nonspecific Neck Pain: A Systematic Review and Meta-Analysis

**DOI:** 10.1155/2017/6197308

**Published:** 2017-08-23

**Authors:** Fushui Liu, Fanyuan Zhou, Meimei Zhao, Ting Fang, Mei Chen, Xiaojun Yan

**Affiliations:** ^1^School of Moxibustion, Jiangxi University of Traditional Chinese Medicine, Nanchang, China; ^2^Research Center for Differentiation and Development of TCM Basic Theory, Jiangxi University of Traditional Chinese Medicine, Nanchang, China

## Abstract

**Objective:**

This review is to assess the efficacy and safety of acupotomy therapy in chronic nonspecific neck pain.

**Methods:**

We searched six computerised databases. Randomized controlled trials incorporating acupotomy therapy alone or combined with other conventional treatments for chronic nonspecific neck pain were included. Two reviewers screened each literature and extracted data independently according to Cochrane Reviews' Handbook (5.1). The Cochrane Collaboration's RevMan 5.3 software was applied for meta-analysis.

**Results:**

A total of ten trials involving 433 patients were enrolled. The pooled analysis indicated that acupotomy therapy showed a significant improving short-term and long-term effect on effective rate and cure rate. Meta-analysis demonstrated that acupotomy therapy group was superior to control group in restoring cervical lordosis and debasing VAS score. The result of continuous data did not support statistical significance of acupotomy therapy in adjusting clinical symptom score. For adverse events, acupotomy group did not reveal obvious superiority compared to control group.

**Conclusions:**

Acupotomy therapy may be beneficial to chronic nonspecific neck pain patients. To strengthen supportive evidence, future, more rigorously designed clinical trials, adequate adverse events, and follow-up project are recommended.

## 1. Introduction

Acupotome is a new-style bladed needle that has a flat head and a cylindrical body, evolving from acupuncture needle [1]. The method of utilizing acupotome to treat soft tissue injuries and bone hyperplasia is given the name, acupotomy therapy [1]. Acupotomy therapy is considered as a minimally invasive surgery of traditional Chinese medicine, combining Chinese acupuncture therapy and modern surgical principles [2]. Acupotomy therapy was first introduced from China in 1976, coming into widespread use. Chinese Academy of Sciences has reported that 360,000 people undergo acupotomy therapy every day [3]. Use of acupotomy therapy saves $8.7 billion compared with surgery and $2.5 billion compared with other treatments, and acupotomy therapy is considered as a safe and effective method [4]. With overseas development of acupotomy therapy, the practitioners of this therapy are living in over thirty countries; meanwhile a book named* Acupotomy Therapy* was translated into five languages and published [3].

Chronic neck pain is defined to be persistent pain or severe discomfort in the neck for over 3 months [5]. About one-half of patients relieve within one year with treatments, but nearly 10% of cases become chronic [6]. Nonspecific neck pain is considered as pain caused by poor posture and mechanical and degenerative changes, excluding pain from neck cancer, infections fasciitis, or other areas of the body [6]. Chronic nonspecific neck pain (CNNP) is a widespread public health issue in the modern time [7]. CNNP ranked 4th highest as for disability and 21st as for overall burden [8]. Hurwitz et al. [9] reported that economic costs of CNNP are estimated to be nearly one hundred of millions of dollars in North Carolina for teachers and state employees in 2009, creating a great financial burden for local residents, families, and government. Lifetime morbidity rate in adults escalates from 14% to 71% [10]. With the change of modern lifestyle and work pattern, CNNP is trending more frequently among adolescents. Based on previous study, the prevalence of CNNP in high school adolescents is 48.9% [11]; a higher prevalence was in women in high-income countries and urban areas, especially in people who are computer engineers or officers [12].

**Figure 1:**
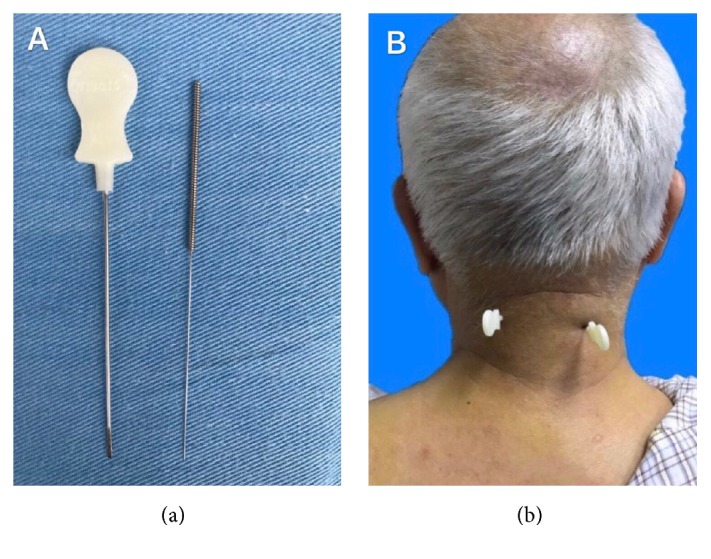
The diagram of the shape of acupotome compared with acupuncture needle (a) and application of acupotomy therapy for CNNP (b).

A systematic review focusing solely on short-term clinical efficacy of acupotomy therapy for treating CNNP concluded that acupotomy is of more benefit than other treatments. In 2012, Liu et al. did a meta-analysis of ten RCTs comparing acupotomy with acupuncture for CNNP; they drew conclusions that acupotomy is superior to acupuncture in terms of short-dated and long-term therapeutic effect [13, 14]. Current systematic reviews only observed short-term efficacy or only assessed clinical efficacy without other secondary outcomes of acupotomy therapy for treating CNNP; we had therefore undertaken a new systematic review of acupotomy therapy for CNNP to identify whether acupotomy has short-term and long-term benefits and to systematically assess secondary outcomes. It showed the shape of acupotome compared with acupuncture needle and application of acupotomy therapy for CNNP in Figure 1.

## 2. Methods

### 2.1. Search Strategy

The following electronic databases were retrieved from their inception until October 19, 2016: PubMed, the Cochrane Library (Issue 4, 2016), Chinese Biomedicine (CBM), the China National Knowledge Infrastructure (CNKI), VIP Information (VIP), and Wanfang Data (WANFANG). We used these search terms: neck pain, chronic non-specific neck pain, neck syndrome, cervical spondylosis, cervical spine, cervical disc, cervical radiculopathy, cervical spondylopathy, acupotomy, acupotome, needle-knife, needle scalpel. CNNP was defined as cervical spondylosis in Chinese, and the same terms in Chinese were searched in Chinese databases. The established search strategy for PubMed was displayed in Table 1.

### 2.2. Inclusion and Exclusion Criteria

Randomized control trials (RCTs) incorporating acupotomy therapy alone or combined with other conventional treatments for CNNP were included. There is no language restriction. The enrolled participants had to be diagnosed definitely with CNNP and no restrictions on age, sex, and duration of illness or source of case. RCTs evaluated clinical effect of acupotomy, compared with no treatment, placebo, or conventional therapies which were considered. Combined therapy of acupotomy and other conventional interventions compared with other conventional interventions in RCTs would also be enrolled. The primary outcome measures included cure rate, effective rate, and adverse effects. The visual analogue scale (VAS), cervical lordosis, and clinical symptom score were assessed as the secondary outcome measures.

The exclusion criteria were shown as follows: (1) no control group; (2) no definite diagnostic criteria of CNNP; (3) wrong interventions: these studies were excluded which used open surgery or acupotomy was manipulated in both groups; (4) duplicated studies; (5) reviews or theory studies; (6) animal experiments.

### 2.3. Document Screening and Data Extraction

Two review authors (Fanyuan Zhou and Meimei Zhao) screened out ineligible studies according to their titles and abstracts independently and then reviewed full text to select the eligible researches. Two authors (Fanyuan Zhou and Meimei Zhao) undertook the extracted data of clinical trials, involving the methodology, interventions, outcomes, follow-up, and withdrawal. Any potential disagreements were resolved by consensus with another team member (Fushui Liu).

### 2.4. Quality Assessment

Methodological quality and risk of bias in included studies were assessed on the basis of Cochrane collaboration's tool [15]: (1) random sequence generation; (2) allocation concealment; (3) blinding of participants and personnel; (4) blinding of outcome assessment; (5) incomplete outcome data; (6) selective reporting; (7) other sources of bias, making a judgment of “low risk of bias,” “unclear risk of bias,” or “high risk of bias” according to the above items. The assessment was carried out by two reviewers (Fanyuan Zhou and Meimei Zhao) independently. Disagreements would be resolved by discussing with the third author (Fushui Liu).

### 2.5. Statistical Treatment

Cochrane Collaboration's Revman 5.3 was applied for meta-analysis. For the continuous data, mean difference (MD) change between two groups was weighted and standardized mean difference (SMD) was selected if different measured methods in different trials were applied to assess the same outcome measure. For categorical data, we calculated combined odds ratio (OR) and 95% confidence interval (CI). Heterogeneity among all studies is based on chi-square test and Higgins *I*^2^ test. We apply random effect model if substantial heterogeneity was detected (*I*^2^ ≥ 50% was regarded as moderate or significant heterogeneity). Otherwise, fixed effect model was employed. Forest plot was generated using Revman 5.3 and funnel plot and Egger's test was prepared to assess publication bias by using Stata 12.0.

## 3. Results

### 3.1. Literature Search Results

**Figure 2:**
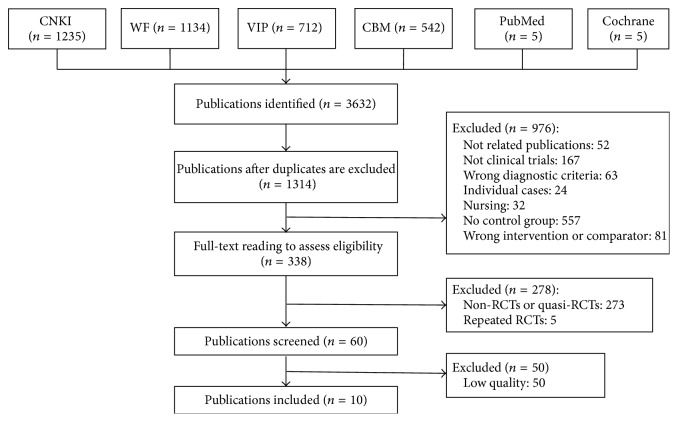
Flow diagram of the trials screening process.

Our research generated 3632 studies, and 1314 studies remained after duplicates were removed. According to our strategy of document screening, 1254 studies were excluded. Then the inferior quality studies were removed and finally 10 suitable RCTs [16–25] were included in the review. The whole process of records screening was shown in Figure 2.

### 3.2. Study Characteristics

The included ten RCTs involve 433 patients with CNNP in acupotomy group and 416 participants in control group. With all but one trial [25] there was multicenter RCT. Sample size was calculated by correct formula in two trials [16, 20], and it was unclear whether the sample size was appropriate in other trials. Three different Chinese acknowledged diagnostic criteria were applied in the ten trials. Only one trial [15] employed self-prepared evaluate criteria; others employed three different criteria in total. In treatment group, four trials [16, 18, 20, 21] used acupotomy therapy and manipulation; three trials [19, 22, 23] used acupotomy therapy in combination with exercise, physiotherapy, traction, or Western medicines. The patients in control group received Western medicines, TCM, acupuncture, traction, manipulation, or physiotherapy. Only three trials [17, 24, 25] employed one single therapy in treatment group and control group. Four trials [17, 19, 20, 23] reported adverse events. The incidence of adverse events of acupotomy group among these four trials was 18.95%, mainly about local pain and bleeding during the treatment. The standard treatment is a course of acupotomy therapy on five to seven continual days, totally for two or three courses. Clinical effective rate and cure rate of acupotomy therapy were calculated in all trials, two of which [19, 25] reported the long-term effective and cure rate after two months and six months, respectively. Two trials referred the recurrence rate [16, 22]; one of them reported that recurrence rate of acupotomy group was 11.32% and that of control group was 24.53% within 1 year. Another trial only reported that the recurrence rate of acupotomy group was 10% within 3 months but without any data about that of control group. Three trials [19, 20, 22] reported VAS score. Three trials measured the cervical lordosis of the treatment group and control group [17, 19, 22]. Four trials reported the clinical symptom score of CNNP [18, 20, 21, 23], while the measured methods of those trials were different. All trials seemed to maintain consistent baseline. Essential characteristics of included trials were summarized in Table 2.

### 3.3. Quality Assessment

**Figure 3:**
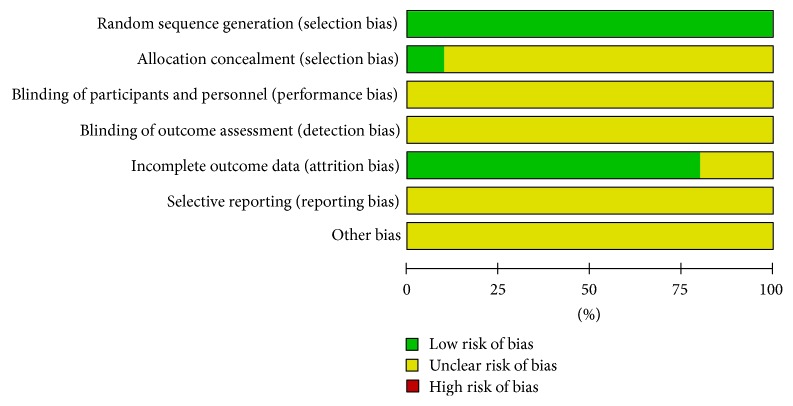
Risk of bias graph.

**Figure 4:**
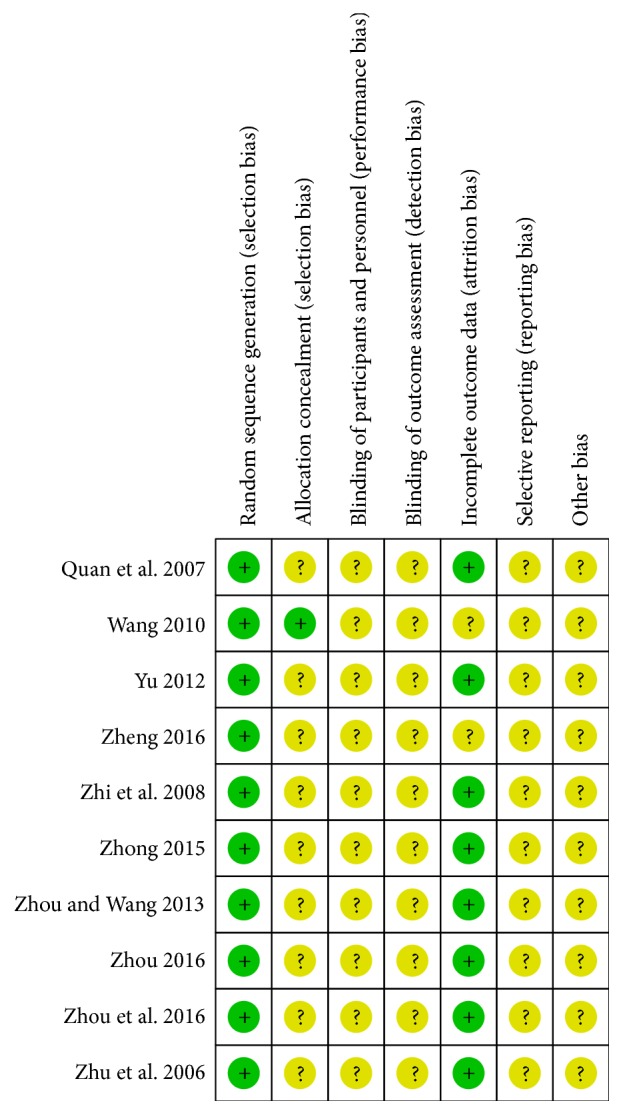
Risk of bias summary.

Quality and risk of bias of included trials were assessed by the Cochrane collaboration's tool. All the included trials reported proper randomization methods, judged to low risk of bias. Two trials [17, 25] employed computer random number generator, one trial [22] used throwing dice, and the others applied random number table. One trial [17] was judged to low risk of bias for using proper opaque envelopes to achieve allocation concealment. Two trials [16, 25] were judged to unclear risk of bias because they just referred assignment envelopes, but it was unknown if the envelopes were sealed. No study reported blinding of participants and personnel, so all trials were judged to unclear risk of bias. When it comes to blinding of outcome assessment, two trials [19, 20] mentioned single blind but it was unclear about the method of blinding; others did not mention any details; thus all trials were judged to unclear risk of bias. Regarding incomplete data, two trials [22, 23] reported no missing data and six trials [16, 18, 21, 23–25] provided the number of dropout and reason for withdrawal. No study referred selective reporting. Estimates of each risk of bias item for all included trials were revealed in Figures 3 and 4.

### 3.4. Clinical Effect

**Figure 5:**
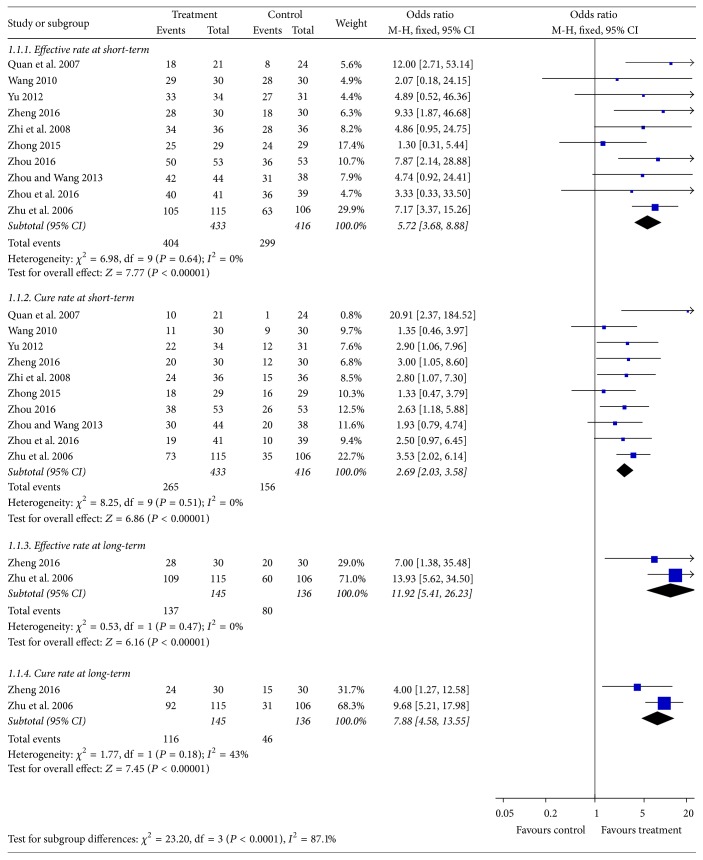
Forest plot of treatment group versus control group: clinical effect.

The four meta-analyses were combined in Figure 5, which demonstrated clinical effect involving effective rate, cure rate, and observation of the two measures at long-term.

#### 3.4.1. Effective Rate at Short-Term

Analysis of data from effective rate at short-term showed no heterogeneity (*I*^2^ = 0%) in all included trials. Results from the pooled data supported the clinical significance of the effective rate of acupotomy group [OR = 5.72; 95% CI = (3.68, 8.88); *Z* = 7.77, *P* < 0.00001].

#### 3.4.2. Cure Rate at Short-Term

No heterogeneity (*I*^2^ = 0%) was detected for cure rate at short-term in all the ten studies. Meta-analysis indicated cure rate of treatment group was higher than that of control group [OR = 2.69; 95% CI = (2.03, 3.58); *Z* = 6.86, *P* < 0.00001].

#### 3.4.3. Effective Rate and Cure Rate at Long-Term

Only two included trials [19, 25] compared acupotomy to other treatments for the outcome of effective rate and cure rate at long-term. The heterogeneity test was confirmed to have no obvious heterogeneity (*I*^2^ = 0%; *I*^2^ = 43%); meta-analysis indicated acupotomy therapy could improve effective rate and cure rate at long-term compared with therapies in control group [OR = 11.92; 95% CI = (5.41, 26.23); *Z* = 6.16, *P* < 0.00001; OR = 7.88; 95% CI = (4.58, 13.55); *Z* = 7.45, *P* < 0.00001].

### 3.5. Cervical Lordosis

**Figure 6:**
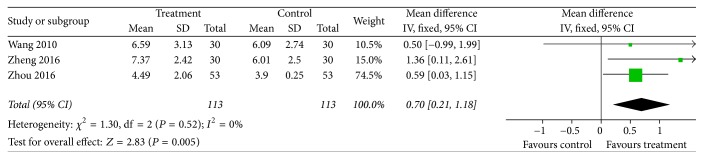
Forest plot of treatment group versus control group: cervical lordosis.

Three trials [17, 19, 22] measured cervical lordosis. Fix effect model was applied with no heterogeneity (*I*^2^ = 0%); the meta-analysis revealed a statistical effect of acupotomy therapy in restoring cervical lordosis [MD = 0.70; 95% CI = (0.21, 1.18); *Z* = 2.83, *P* = 0.005] (Figure 6).

### 3.6. VAS Score

**Figure 7:**
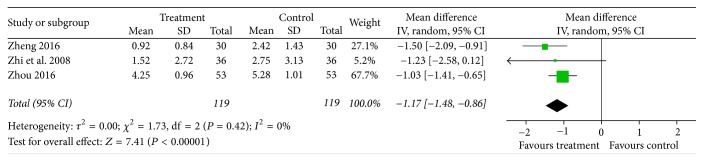
Forest plot of treatment group versus control group: VAS score.

Three trials [19, 20, 22] reported VAS score to measure pain intensity. No heterogeneity was found (*I*^2^ = 0%); the meta-analysis indicated acupotomy therapy helped to debase VAS score compared with therapies in control group [MD = −1.00; 95% CI = (−1.30, −0.70); *Z* = 7.41, *P* < 0.00001] (Figure 7).

### 3.7. Clinical Symptom Score

**Figure 8:**
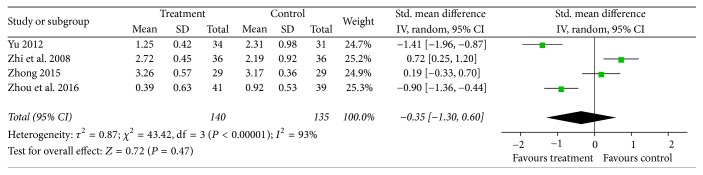
Forest plot of treatment group versus control group: clinical symptom score.

Random effects model was used, because significant heterogeneity existed (*I*^2^ = 93%) in continuous data for clinical symptom score from four studies [18, 20, 21, 23] which observed clinical symptom score. The obvious heterogeneity could be ascribed to the different measured methods in different studies. Patients were asked to grade diverse clinical symptoms according to their own pain intensity: no pain, feeling sometimes pain, often but not serious pain, and always serious pain. In Zhi's and Zhong's studies, 0 points were awarded if patients always feel serious pain; if they felt no pain they scored 3 points, so higher points were favourable for treatment group, while other two trials were graded on a contrary scoring criteria. We had therefore chosen SMD for analyzing the data. The combined data showed no significant difference in improving clinical symptoms between acupotomy and other conventional treatments [SMD = −0.35; 95% CI = (−1.30, 0.60);* Z* = 0.72, *P* = 0.47] (Figure 8).

### 3.8. Adverse Events

**Figure 9:**
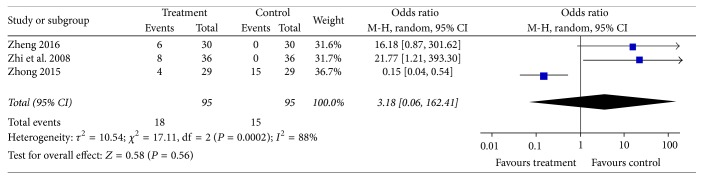
Forest plot of treatment group versus control group: adverse events.

Only three trials [19, 20, 23] reported specific number of adverse events. Another trial [15] referred adverse events without the number. Random effects model was utilized with biggish heterogeneity (*I*^2^ = 88%). Meta-analysis showed no statistical significance in adverse events recorded between the two groups [OR = 3.18; 95% CI = (0.06, 162.41); *Z* = 0.58, *P* = 0.56] (Figure 9). Adverse events in acupotomy therapy group accounted for about 18.95% among all included patients and mainly about local pain and mild bleeding, indicating acupotomy therapy was safe in some degree, but it remained unclear whether acupotomy therapy was safer than other conservative treatments.

### 3.9. Publication Bias

**Figure 10:**
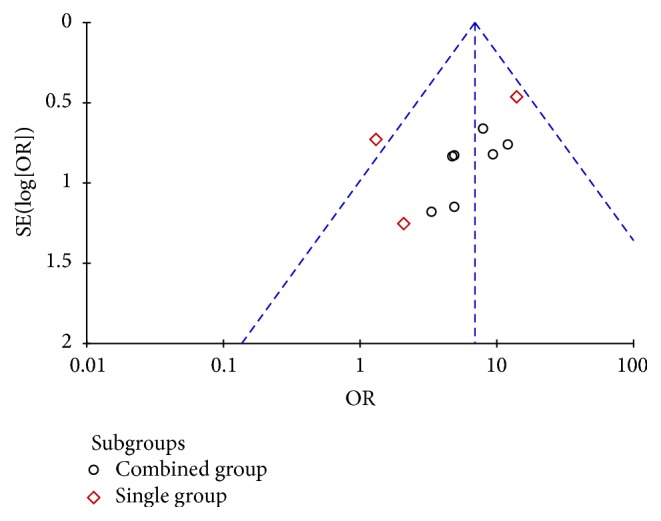
Funnel plot of treatment group versus control group: effective rate.

**Figure 11:**
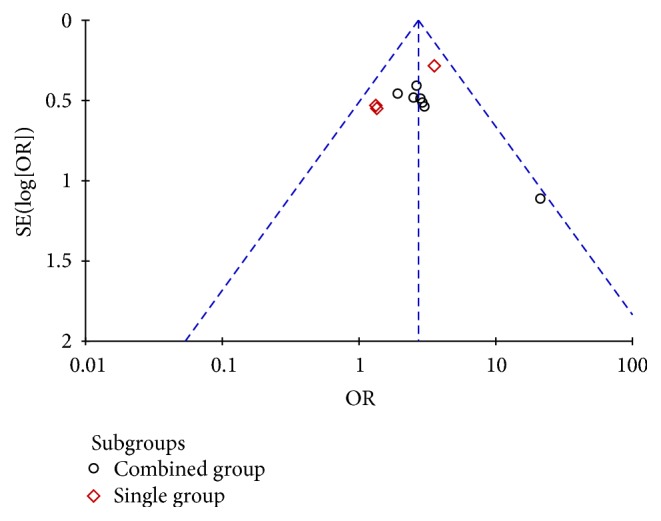
Funnel plot of treatment group versus control group: cure rate.

Funnel plots were applied to estimate publication bias (Figures 10 and 11). The graph showed moderate asymmetry for effective rate, while there seemed to be no obvious asymmetry for cure rate. Furthermore, Egger's test indicated no statistical significance of publication bias (effective rate: *t* = −0.20, *P* = 0.845; cure rate: *t* = 0.92, *P* = 0.382).

## 4. Discussion

Chronic nonspecific neck pain is generated by any structures in the neck involving muscle, bone, vascular, and nerve and often induces disability to work [26]. The common causes of CNNP include neck strain, physical or emotional stress, prolonged inappropriate postures, minor injuries or falls, over-use, and herniated intervertebral discs [27]. CNNP relieves pain with nonsurgery treatments such as medicines, exercise plus joint mobilization, cervical manipulation, acupuncture, or acupotomy and becomes immedicable in only about 5%–10% of patients [28, 29]. And a systematic review showed that the efficacy of surgery over other conservative treatments is not clearly confirmed [30].

Acupotomy is considered as aggressive therapy, using a knife-shaped needle tip to peel inside the damaged soft tissues [1]. The theory of acupotomy therapy suggests that strain and adhesions caused at cervical muscles and soft tissue will disturb dynamic equilibrium of cervical vertebrae, which lead to cervical diseases [31]. Chronic soft tissue injury as a main cause of CNNP is the indispensable process [29]. Acupotomy therapy is beneficial in removing adhesions, scar, contractures, and relieving tension of soft tissue to restore dynamic equilibrium of neck [32]. Many animal experiment studies indicated that the mechanism of acupotomy therapy may involve restoring cervical lordosis, improving the local microcirculation, providing analgesic effect, and reducing inflammatory factors and cervicomuscular cellular apoptosis [33–37].

Acupotomy has come to be widely used in the treatment of CNNP [38, 39]. However, evidence to assess the long-term efficacy of acupotomy and secondary outcomes for CNNP is scarce. Categorical data for clinical effect significantly favoured acupotomy at both short-term and long-term for treating CNNP. Meta-analysis indicated that acupotomy helps restore cervical lordosis and relieve the pain, but it still needs to be proved with larger sample size. For clinical symptom score, the combined data showed no significant difference between acupotomy group and control group. The difference of adverse events between two groups did not reach what is generally considered the minimally clinically important difference. Many experts suggested it is considered safe if the practitioners were equipped with knowledge of anatomical structures [40, 41], and clinical adverse events mainly focus on slight bleeding and local pain during the treatment. We therefore did not determine the adverse events in the course of acupotomy so far. Latest research reported a safer method [42] that acupotomy therapy can be visible with ultrasound guidance, reducing the risk of blind sight of traditional acupotomy therapy. Perhaps visualization of acupotomy therapy is a trend and well worth for further investigation.

Nevertheless, our study had several limitations as follows: unable to assess racial difference in effect of acupotomy therapy because all the included trials were published in Chinese. All included trials estimated “cure,” “effective,” and “ineffective” by the feeling of participants but not quantitative standard; it remained debatable. Regarding secondary outcome measures, the measurement criteria were diverse, resulting in definite conclusion not being drawn. Only a handful of included trials reported allocation concealment, blinding of outcome assessment, and follow-up observation. Small number of enrolled trials recorded adverse events. In view of the above defect, more scientific clinical trials, adequate adverse events, and follow-up design are recommend.

## 5. Conclusions

According to our study, acupotomy therapy has short-term and long-term benefits for chronic nonspecific neck pain, and it helps alleviate pain and restore cervical lordosis. While it remains to be further researched whether acupotomy is of benefit to improve the clinical symptoms. Our result does not provide strong evidence for safety of acupotomy therapy. To strengthen supportive evidence, future, more rigorously designed clinical trials, adequate adverse events, and follow-up project are recommend.

## Figures and Tables

**Table 1:** Search strategy for PubMed.

Number	Search terms
1	Mesh term: ((acupotomy) or (acupotome) or (needle knife) or (needle scalpel)): ti, ab, kw
2	Mesh terms: ((chronic non-specific neck pain) or (neck pain) or (neck syndrome) or (cervical spondylosis) or (cervical spine) or (cervical disc) or (cervical radiculopathy) or (cervical spondylopathy)): ti, ab, kw
3	Mesh terms: ((clinical trials) or (random control trials))
4	1 and 2 and 3

**Table 2:** Essential characteristics of included trials.

Study	Population (T/C)	Intervention		Outcome	Duration	Adverse events
		Treatment	Control			
Quan et al. 2007 [16]	21/24	Acupotomy + manipulation	WM	CE	20 days	NR
Wang 2010 [17]	30/30	Acupotomy	Electroacupuncture	CE, CL	21 days	Yes
Yu 2012 [18]	34/31	Acupotomy + manipulation	TCM	CE, CSS	20 days	NR
Zheng 2016 [19]	30/30	Acupotomy + exercise	WM + exercise	CE, CL, VAS	21 days	Yes
Zhi et al. 2008 [20]	36/36	Acupotomy + manipulation	Traction	CE,CSS, VAS	20 days	Yes
Zhou et al. 2016 [21]	41/39	Acupotomy + manipulation	Manipulation	CE, CSS	14 days	NR
Zhou 2016 [22]	53/53	Acupotomy + traction + WM	Traction + WM	CE, CL, VAS	21 days	NR
Zhou and Wang 2013 [23]	44/38	Acupotomy + PT	Acupuncture + PT	CE, CSS	14 days	NR
Zhong 2015 [24]	29/29	Acupotomy	WM	CE	14 days	Yes
Zhu et al. 2006 [25]	115/106	Acupotomy	Acupuncture	CE	20 days	NR

*Note*. NR: not reported; WM: Western medicines; TCM: traditional Chinese medicine; PT: physiotherapy; CE: clinical effect; CSS: clinical symptom score; CL: cervical lordosis; VAS: visual analogue scale.
